# Effects of *Allium macrostemon* Bunge Extract on Adipose Tissue Inflammation and Hepatic Endoplasmic Reticulum Stress in High-Fat Diet-Fed and Bisphenol A-Treated C57BL/6N Mice

**DOI:** 10.3390/foods12203777

**Published:** 2023-10-14

**Authors:** Juhae Kim, Na-Hyung Kim, Isoo Youn, Eun Kyoung Seo, Choon Young Kim

**Affiliations:** 1 Research Institute of Human Ecology, Yeungnam University, Gyeongsan 38541, Republic of Korea; kimjh825@yu.ac.kr (J.K.); knahyu@yu.ac.kr (N.-H.K.); 2 Graduate School of Pharmaceutical Sciences, College of Pharmacy, Ewha Womans University, Seoul 03760, Republic of Korea; isooyoun87@gmail.com (I.Y.); yuny@ewha.ac.kr (E.K.S.); 3 Department of Food and Nutrition, Yeungnam University, Gyeongsan 38541, Republic of Korea

**Keywords:** Bisphenol A, high-fat diet, *Allium macrostemon* Bunge, adipose tissue, inflammation, liver

## Abstract

The simultaneous exposure to a high-fat (HF) diet and to bisphenol A (BPA) from delivered foods and food-delivery containers is on the rise in humans, according to the increased frequency of food delivery during the COVID-19 pandemic. This co-exposure could cause harmful tissue toxicity in the human body. Here, the preventive effect of *Allium macrostemon* Bunge (AM) extract against dysfunction in adipose tissue and the liver under co-exposure to BPA and an HF diet was examined using mice. C57BL/6N mice were divided into four groups (*n* = 6 or 7/group) according to diet and treatment: control diet with vehicle (CON), HF diet with vehicle (HF), HF diet with an oral injection of BPA (HF + BP), and HF diet with an oral injection of BPA and AM extract (HF + BP + AM). HF feeding increased body weight gain compared to CON feeding, while BP + HF and BP + HF + AM feeding suppressed body weight gain compared with HF feeding. The BP + HF group had lower body weight than the HF group, but the two groups had similar epididymal fat mass. The HF + BP + AM group showed lower pro-inflammatory gene expression levels in adipose tissue and epididymal fat mass compared to the HF + BP group. Altered endoplasmic reticulum (ER) stress response in the liver was partly observed in the HF + BP group, as shown by increased total phosphorylated Jun N-terminal kinase protein levels compared to those in the HF group. In addition, ecdysterone 25-O-β-D-glucopyranoside and 6-gingerol were identified in AM extract by mass spectrometry and molecular networking analysis. In summary, the AM extract diminished adipose tissue inflammation and hepatic ER stress in an HF diet and BPA co-exposure condition. To utilize AM as a potential food component to alleviate the harmful effect of an HF diet and BPA exposure, further research investigating the specific impact of AM extract supplementation using additional experimental groups or various treatment doses is warranted.

## 1. Introduction

Food delivery services surged due to social distancing during the coronavirus disease 2019 (COVID-19) pandemic [1]. Representative delivery menus include foods containing high amounts of fat, which are frequently consumed in westernized diets. The fat in foods reportedly eases the migration of bisphenol A (4,4′-isopropylidenediphenol, BPA) particles to food, resulting in increased exposure of humans to BPA [2]. BPA is a frequently used compound in the production of polycarbonate plastics and epoxy resin. Due to its structural similarity to estrogen, BPA is classified as an endocrine-disrupting chemical with estrogenic activity. BPA is known to have negative health effects related to reproductive function, immune function, and metabolic function [2,3]. Notably, these health conditions can also be influenced by the consumption of a high-fat (HF) diet. In keeping with this trend, several studies on co-exposure have demonstrated the negative effects of a combined HF diet and BPA exposure on metabolic health.

The elevation of inflammation in adipose tissue is reported to be a consequence of BPA exposure when added to HF conditions [4]. The release of inflammatory adipokines and excessive amounts of free fatty acids (FFAs) are characteristics of dysfunctional adipose tissue [5]. Interleukin (IL)-1β, tumor necrosis factor-α (TNF-α), F4/80, and monocyte chemoattractant protein-1 (MCP-1) are examples of pro-inflammatory adipokines. Immune cell infiltration, histologically detected by a crown-like structure (CLS), is also a prominent feature of dysfunctional inflamed adipose tissue [6]. FFAs promote ectopic fat deposition and lipotoxicity, of which a representative molecular mechanism is endoplasmic reticulum (ER) stress in the liver. In addition to this secretory function, adipose tissue has been suggested as a potential site of toxicant accumulation [7]. Consistently, susceptibility of adipose tissue to BPA has resulted in its being reported as the site containing the highest amount of free BPA concentrations, followed by the liver and brain, as per a human study [8].

The synergistic harmful effects of BPA and an HF diet on the liver have also been reported as chronic ER stress-mediated steatosis progression [9] and immune-metabolic dysfunction [10]. The role of ER stress in hepatic dysfunction has been well demonstrated, as BPA exposure increased ER stress-associated apoptosis or hepatocellular injury in in vitro as well as in vivo models [9,11]. During ER stress, the unfolded protein response involves activation of transmembrane ER-resident stress sensors, which involves sequestering and binding of the chaperone, binding immunoglobulin protein (BiP), to unfolded proteins [12]. One sensor is inositol-requiring kinase 1 (IRE1), which subsequently activates Jun N-terminal kinase (JNK), an inducer of cell apoptosis and inflammation [12]. Another transducer, protein kinase RNA-like endoplasmic reticulum kinase (PERK), is activated by the phosphorylation of eukaryotic initiation factor 2 alpha (eIF2α) and translation of activating transcription factor 4 (ATF4). The transcription of C/EBP homologous protein (CHOP), known as the downstream target of ATF4, is a well-characterized proapoptotic pathway derived from the stressed ER [12]. The eIF2α/ATF4 pathway has also been reported to induce the transcription of genes involved in autophagy, such as *Becn 1* and *Ma1lc3b*, which encode autophagy marker proteins beclin 1 and microtubule-associated protein light chain 3 (LC3) [13].

The therapeutic properties of many nutraceuticals against the adverse health effects of BPA per se have been investigated [14]. Resveratrol [15], lycopene [16], curcumin [17], and thymoquinone [18] ameliorate BPA-induced metabolic disorders, including hyperlipidemia, upregulated inflammation, and insulin resistance. In addition to single compounds, food extracts from olive leaves, green tea, grape seeds, and ginger have been reported to possess a protective role against BPA-induced damage, including liver injury and lipid metabolism disturbance [19], vascular toxicity [20,21], and metabolic syndrome [15]. In line with these findings, it has been reported that plants have an intrinsic detoxification potential for reducing BPA content through oxygenation to quinones using polyphenol oxidase [22].

*Allium macrostemon* Bunge (AM), one of the plant species included in the Chinese Pharmacopoeia [23], is commonly grown in East Asia [24] and has shown a variety of beneficial effects, including lipid-lowering, antioxidant, and anti-obesity activities, in various study models, including in vitro, in vivo, and clinical systems [25,26,27,28,29]. Previously, we reported that AM whole extract (200 mg/kg BW daily) treatment ameliorates inflammation and endoplasmic reticulum stress in adipose tissue of HF diet-induced C57BL/6N mice [29]. Here, we hypothesized that the simultaneous exposure to HF and BPA from delivery containers and delivery foods in humans would be higher than before, as the delivery food culture increased rapidly as a result of COVID-19. Since we assumed that main targeting organs for BPA and HF co-exposure are the adipose tissue and liver, this study aimed to evaluate the effect of AM on adipose tissue and liver dysregulation, focusing on inflammation and ER stress, respectively.

## 2. Materials and Methods

**Figure 1:**
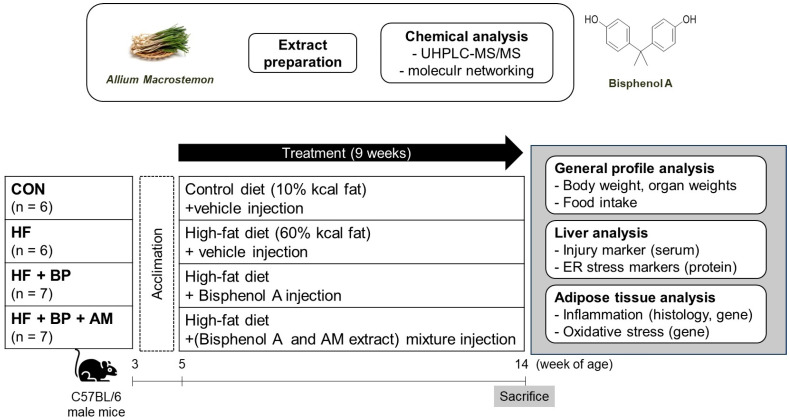
Flow chart for experiments. Information for treatment compounds (preparation and analysis for *Allium macrostemon* extract and chemical structure of bisphenol A) are indicated in the upper figure. In the bottom figure, the animal experimental scheme is presented. The specific analysis topic is presented in the gray box. CON, control diet group; HF, high-fat diet group; HF + BP, high-fat diet and bisphenol A–treated group; HF + BP + AM, high-fat diet and bisphenol A plus *Allium macrostemon* extract–treated group.

The Flow chart representing the whole experimental design is presented in Figure 1.

### 2.1. Allium macrostemon Bunge (AM) Preparation and Chemical Analysis

#### 2.1.1. Preparation of AM Extract

AM was purchased from a commercial market (Sacheon, Gyeongnam, Republic of Korea) in April 2021. As previously reported [28], the dried part of the AM was extracted using a high-pressure/high-temperature reactor at 118 kPa/121 °C for 20 min. After filtration, the solvent was removed, and the extracts were concentrated using a rotary evaporator and lyophilized. The yield was 54.8%. The obtained AM extract was stored at −20 °C until use.

#### 2.1.2. Mass Spectrometry Data Acquisition for Chemical Analysis of AM Extract

Ultra-high-performance liquid chromatography tandem mass spectrometry (UHPLC-MS/MS) data acquisition was conducted on a Waters SYNAPT G2-Si quadrupole time-of-flight mass spectrometer with MassLynx 4.1 software (Waters, MA, USA). Separation of the AM extract was achieved on an Agilent Zorbax Eclipse Plus C18 column (2.1 × 150 mm, 1.8 μm, Agilent Technologies, Santa Clara, CA, USA). The mobile phase was water with 0.1% formic acid (A) and acetonitrile with 0.1% formic acid (B). The gradient elution was as follows: 0–2.5 min, 5% B; 2.6–21 min, 5–100% B; 21–26 min, 100% B; 26.1–30 min, 5% B. The column oven was set to 40 °C, and the autosampler temperature was 15 °C. The flow rate was 0.4 mL/min, and the injection volume was 1 μL. Comprehensive mass spectra information was acquired using a negative MS^E^ mode. The parameters of the MS^E^ mode were set as follows: mass range, 100–1200 Da; capillary voltage, 2.2 kV; sampling cone voltage, 50 eV; source offset voltage, 30 eV; source temperature, 120 °C; desolvation temperature, 450 °C; cone gas flow rate, 50 L/h; and desolvation gas flow rate, 800 L/h. Nitrogen and argon were applied as cone and collision gases, respectively. The collision energy was 20–40 eV for high energy function, and the scan time was 0.5 s. The data were calibrated in real time using a leucine-enkephalin solution (*m*/*z* 554.2615 [M-H]^−^) as an external reference (LockSpray™) at a flow rate of 5 μL/min.

#### 2.1.3. Feature-Based Molecular Networking Analysis of AM Extract

The acquired data from UHPLC coupled with a quadrupole time-of-flight mass spectrometer was analyzed for feature-based molecular networking (FBMN) based on the online FBMN-Progenesis QI workflow (https://ccms-ucsd.github.io/GNPSDocumentation/featurebasedmolecularnetworking-with-progenesisQI/ (accessed on 1 May 2023) [30]. The obtained MS data were processed using Progenesis QI software (v3.0) for alignment and peak-picking (intensity > 10,000; spectral width > 0.1 min). Both the feature quantification table (CSV file) and MS/MS spectral fragment summary (MSP file) of the raw LC-MS/MS data were exported from the Progenesis QI and submitted to the GNPS platform (https://gnps.ucsd.edu (accessed on 1 May 2023)) using WinSCP (https://winscp.net (accessed on 1 May 2023)). The configurations of network parameters were as follows: precursor ion mass tolerance and fragment ion mass tolerance, 0.02; minimum cosine score, 0.70; minimum matched fragment ions, 6; maximum number of neighbor nodes for one single node, 10; and maximum size of a spectral family, 100. Cytoscape (ver. 3.9.1) was used for network visualization and analysis.

### 2.2. Animal Experiment

#### 2.2.1. Animals and Experimental Design

Three-week-old male C57BL/6N mice were obtained from Central Lab Animal (SLC, Osaka, Japan) and housed under optimal environmental conditions at a temperature of 21 ± 2 °C and a humidity of 50 ± 5% under a 12:12 light:dark cycle. After acclimation for two weeks, the mice were randomly divided into four groups, as follows:

(i) the control (CON) group fed a control diet (*n* = 6);
(ii) the high-fat (HF) group fed an HF diet (*n* = 6);
(iii) the HF + BP group fed an HF diet and treated with BPA (*n* = 7);
(iv) the HF + BP + AM group fed an HF diet and treated with BPA and AM extract (*n* = 7).

The control diet (containing 10 kcal% fat, 20 kcal% protein, and 70 kcal% carbohydrate, D12450B) and the HF diet (containing 60 kcal% fat, 20 kcal% protein, and 20 kcal% carbohydrate, D12452) were purchased from Research Diets, Inc. (New Brunswick, NJ, USA) and provided ad libitum. BPA and/or AM extracts were dissolved in distilled water containing 0.01% dimethyl sulfoxide (Sigma-Aldrich Co., St. Louis, MO, USA). The mice in the CON and HF groups were administered a vehicle solution by oral gavage. The HF + BP group was treated with 50 μg/kg/day of BPA. The HF + BP + AM group was treated with 50 μg/kg/day of BPA plus 400 mg/kg/day of AM extract simultaneously. The treatment or vehicle solutions were administered daily via oral gavage (10 μL/g body weight) based on the measured body weight, neglecting daily body weight changes. During the experimental period of 9 weeks, body weight and food intake were measured twice or three times per week. Energy intake was calculated by multiplying food intake by the respective energy content of each diet (3.85 kcal/g for the CON group and 5.24 kcal/g for other groups fed an HF diet). All animal procedures were performed according to protocols approved by the Institutional Animal Care and Use Committee of Yeungnam University (approval number: #2021-033).

#### 2.2.2. Serum and Tissue Isolation

After the experimental treatment for 9 weeks, all mice were fasted for 16 h and anesthetized with isoflurane (Isotroy 250; Troikaa Pharmaceuticals, Gujarat, India). Before tissue harvesting, blood was collected by cardiac puncture, transferred into serum separation tubes (BD Microtainer ^®^ tube, #365967, BD Biosciences, Franklin Lakes, NJ, USA), clotted for 30 min at RT, and centrifuged at 15,000× *g* rpm at 4 °C for 90 s. Isolated serum was collected and analyzed for liver injury biomarkers (glutamic oxaloacetic transaminase (GOT) and glutamic pyruvic transaminase (GPT)) and kidney injury biomarkers (blood urea nitrogen (BUN)) (Asan Pharmaceutical Co., Seoul, Republic of Korea), using commercial colorimetric kits according to the manufacturer’s instructions. Absorbance was measured at 505 nm for GOT and GPT and 580 nm for BUN using a SpectraMax ID3 microplate reader (Molecular Devices, San Jose, CA, USA). Epididymal, perirenal, and retroperitoneal fat pads as well as liver tissue were rapidly removed from the mice and weighed immediately. The tissues were snap-frozen in liquid nitrogen and stored at −70 °C until analysis.

#### 2.2.3. Histological Analysis for Adipose Tissue

Epididymal adipose tissues were fixed in 10% neutral formalin solution (#HT501128; Sigma-Aldrich Co.) and embedded in paraffin. The tissues were cut to a thickness of 6 μm and stained with hematoxylin and eosin (H&E). Using a light microscope (Eclipse Ni-U, Nikon, Tokyo, Japan), the number of CLSs in each stained section from four different mice in each group was manually counted and recorded. For each slide, the analysis was performed in four randomly selected fields (100× magnification). The total number of CLSs was expressed as the mean number per field.

#### 2.2.4. RNA Extraction, cDNA Synthesis, and Real-Time Polymerase Chain Reaction for Adipose Tissue

Epididymal adipose tissues were homogenized using the TissueLyser system (#85300, Qiagen, Venlo, The Netherlands), and total RNA was isolated using TRIzol reagent (#15596018, Invitrogen Life Technologies, Carlsbad, CA, USA) according to the manufacturer’s instructions. RNA content and purity were measured using a NanoDrop spectrophotometer (Thermo Fisher Scientific, Waltham, MA, USA). One microgram of total RNA was reverse-transcribed into cDNA using the AMPIGENE^®^ cDNA synthesis kit (#END-KIT106, Enzo Life Sciences, Farmingdale, NY, USA) and a SimpliAmp Thermal Cycler (Applied Biosystems, Waltham, MA, USA) according to the manufacturer’s instructions. Real-time PCR was performed using AMPIGENE^®^ qPCR Green Mix Hi-ROX (#ENZ-NUC104, Enzo Life Sciences) on a StepOne Plus real-time PCR system (Applied Biosystems). The thermal cycles were as follows: 94 °C for 3 min, followed by 40 cycles at 95 °C for 10 s, 60 °C for 15 s, and 72 °C for 20 s. Mouse 18s rRNA was used as a reference gene, and relative gene expression levels were analyzed using the 2^−ΔΔCt^ method. The primers used for the real-time PCR analysis are listed in Supplementary Materials Table S1.

#### 2.2.5. Protein Extraction and Western Blotting Analysis for Liver Tissue

Liver tissues were homogenized in ice-cold lysis buffer (100 mM Tris-HCl, pH 7.6, 100 mM NaCl, and 0.5% Triton X-100) containing 1 mM sodium orthovanadate, 10 mM sodium fluoride, and a protease inhibitor cocktail (#P3100, GenDEPOT, Katy, TX, USA) using the TissueLyser system (#85300, Qiagen). The liver homogenates were incubated at 4 °C for 20 min with gentle rotation and centrifuged at 10,000× *g* for 30 min at 4 °C. The epididymal fat homogenates were incubated at 4 °C for 1 h with gentle rotation and centrifuged three times at 20,000× *g* for 15 min at 4 °C to remove excess lipids. The final supernatant from each tissue sample was collected and stored at −70 °C until further analysis. The protein content of the lysates was determined using the Bradford protein assay kit (Bio-Rad, Hercules, CA, USA). Equal amounts of protein were loaded onto the lanes of a sodium dodecyl sulfate-polyacrylamide gel electrophoresis gel, separated, and transferred onto a polyvinylidene difluoride membrane (Millipore, Bedford, MA, USA). After blocking with 5% non-fat milk (BD Biosciences) or bovine serum albumin (Bovostar, Bovogen, Victoria, Australia) in a Tris-buffered saline solution containing 0.05% Tween-20 (pH 7.5), the membrane was probed with a specific primary antibody at 1:1000 dilution of one of the following: ATF4 (#11815, Cell Signaling Technology, Danvers, MA, USA), beclin-1 (#3495, Cell Signaling Technology), phosphorylated eIF2α (#3398, Cell Signaling Technology), eIF2α (#9722, Cell Signaling Technology), heat shock cognate 70 (HSC70; #sc-7298, Santa Cruz Biotechnology, Dallas, TX, USA), phosphorylated IRE1α (#NB-100-2323, Novus Biologicals, Centennial, CO, USA), IRE1α (sc-390960, Santa Cruz Biotechnology), phosphorylated JNK (#4668, Cell Signaling Technology), JNK (#9252, Cell Signaling Technology), LC3B (#2775, Cell Signaling Technology), and SQSTM1/p62 (#5114, Cell Signaling Technology). The membrane was then incubated with anti-mouse or anti-rabbit horseradish peroxidase-linked secondary antibody (Jackson ImmunoResearch, West Grove, PA, USA). The protein expression signal was detected using the Amersham ECL western blotting Detection Reagent (GE Healthcare, Piscataway, NJ, USA). Immunoreactive bands were visualized and analyzed using Amersham ImageQuant 800 (Cytiva, Marlborough, MA, USA). The expression of HSC70 was used as a control to monitor equal protein loading in each lane.

### 2.3. Statistical Analysis

Data are presented as mean ± standard error of the mean (SEM). For serum parameters, outliers identified using Grubbs’ outlier test were excluded from the analyses, as previously reported [29]. The statistical analysis was conducted with one-way analysis of variance (ANOVA) followed by a Tukey post hoc test for multiple comparison. Significance was determined at a *p*-value of less than 0.05. All statistical analyses were carried out using SPSS 25 (IBM, Chicago, IL, USA). A minimum sample size of six mice per group was based on the ability to detect a minimal meaningful difference to provide an α = 0.05 and a power of 80%. Calculation of the sample size was performed using G*Power Software (version 3.0.10, Germany).

## 3. Results

### 3.1. Effects of AM Supplementation on Body Weight Gain, Energy Intake, Organ Weights, and Serum Analysis

During the 9-week experimental period, HF feeding induced a significant increase in body weight compared to that in the CON diet feeding (Table 1). The body weight gain in the HF + BP group was significantly lower by 1.3-fold than that in the HF group. No further effect of AM supplementation on body weight gain was observed. Energy intake was 1.3-fold higher in both the HF group and the HF + BP group than that in the CON group (Table 1). Similar to the body weight changes, the weights of the epididymal fat, kidneys, and spleen were 3.6-fold, 1.2-fold, and 1.2-fold higher, respectively, than those of the CON group (Table 1). Notably, the epididymal fat weight of the HF + BP + AM group was significantly decreased by 1.3-fold compared to that of the HF + BP group. A similar pattern was observed in the case of the sum of the perirenal and retroperitoneal fat weights. The spleen weight was lower in the HF + BP group than in the HF group, with no further effect of AM supplementation. Liver and kidney injury biomarkers were also evaluated as indicated in Table 1. Although there were no significant alterations in biomarker levels among all groups, we observed a 1.51-fold increase in GPT levels in the HF + BP group compared to that in the HF group.

### 3.2. Effects of AM Supplementation on Adipose Tissue Inflammation and Oxidative Stress

**Figure 2:**
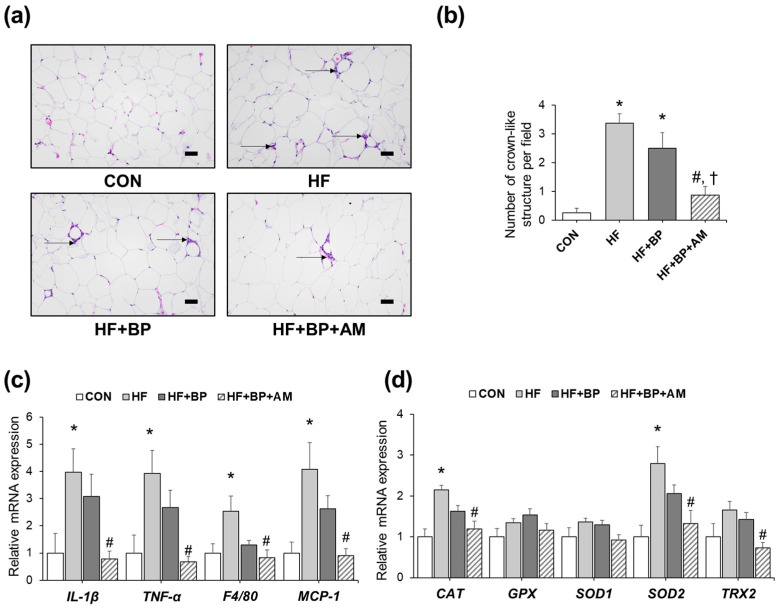
Effects of *Allium macrostemon* (AM) extract supplementation on adipose tissue inflammation and oxidative stress in high-fat diet-treated and BPA-treated mice. (**a**) Representative hematoxylin and eosin (H&E) staining of epididymal adipose tissue (200× magnification, scale bar = 50 μm) is shown. Crown-like structures (CLSs) are depicted with arrows. (**b**) The number of CLSs was counted under one field in 100× magnification. (**c**) Relative mRNA levels of pro-inflammatory genes were measured by real-time PCR. (**d**) Relative mRNA levels of antioxidant enzyme genes were measured by real-time PCR. Data are expressed as mean ± SEM (*n* = 4 for histological analysis, *n* = 5–6 for gene expression analysis). * *p* < 0.05 versus CON; # *p* < 0.05 versus HF; † *p* < 0.05 versus HF + BP. CON, control diet group; HF, high-fat diet group; HF + BP, high-fat diet and bisphenol A–treated group; HF + BP + AM, high-fat diet and bisphenol A plus *Allium macrostemon* extract–treated group.

Since there were significant changes in adipose tissue weight among the groups (Table 1), histological and molecular changes were examined by H&E staining and qRT-PCR analysis in adipose tissue. The number of CLSs, a hallmark of macrophage infiltration, was markedly increased in the HF group as well as in the HF + BP group by 13.5-fold and 10.0-fold, respectively, compared to that in the CON group (Figure 2a,b). Notably, the HF + BP + AM group showed significantly lower levels of CLS than the HF + BP group, by 2.86-fold. Similar to histological observations, the mRNA expression levels of pro-inflammatory markers, including IL-1β and TNF-α, and macrophage markers, including F4/80 and MCP-1, were higher in the HF group than in the CON group. Similarly, slightly lower levels of these pro-inflammatory markers were observed in the HF + BP group than in the HF group (Figure 2c). Significantly lowered levels of inflammatory gene expression were detected in the HF + BP + AM group compared to the HF group. Although there was a decreasing tendency, no significant differences between the HF + BP + AM group and HF + BP group were determined. Changes in the expression of antioxidative enzymes were also evaluated, as shown in Figure 2d. The gene expression levels of catalase (CAT), superoxide dismutase 2 (SOD2), and thiredoxin2 (TRX2) were higher in the HF group than in the CON group. The HF + BP group showed a similar or lower tendency of expression levels compared to those in the HF group. Significantly lowered levels of three of antioxidative enzymes’ gene expression (CAT, SOD2, TRX2) were detected in the HF + BP + AM group compared to the HF group. AM supplementation lowered the gene expression levels of SOD2 and TRX2 by 1.56-fold and 1.95-fold, respectively, compared to those of the HF + BP group.

### 3.3. Effects of AM Supplementation on Hepatic ER Stress and Autophagy

**Figure 3:**
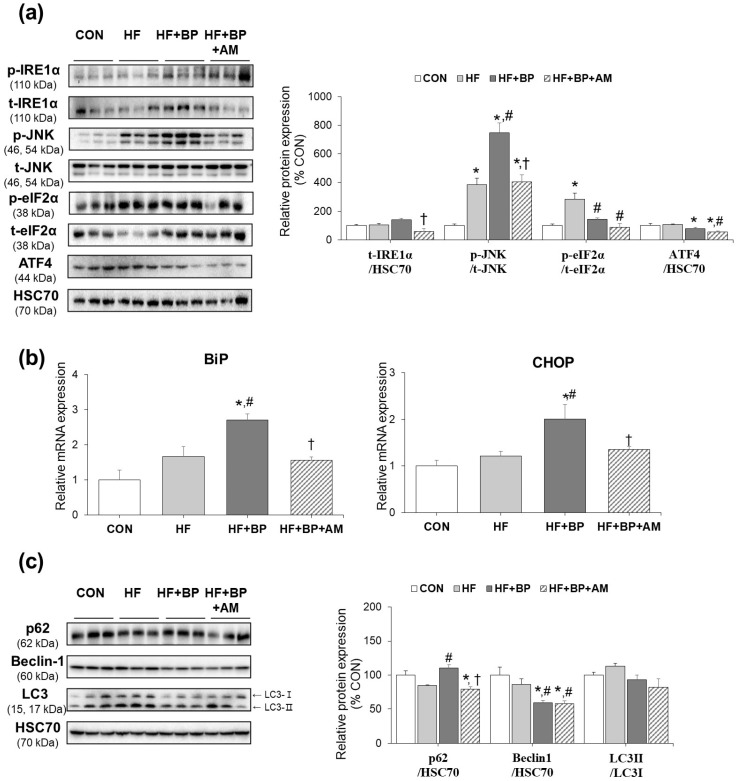
Effects of *Allium macrostemon* (AM) extract supplementation on hepatic endoplasmic reticulum (ER) stress and autophagy in high-fat diet-treated and BPA-treated mice. (**a**) Protein expression of ER stress-related mediators, including total IRE1α, phosphorylated and total JNK, phosphorylated and total eIF2α, and ATF4 was measured by Western blot analysis. (**b**) Relative mRNA levels of ER stress-related mediators, including BiP and CHOP, were measured by real-time PCR. (**c**) Protein expression of autophagy-related mediators, including p62, beclin-1, and LC3, were determined by Western blot analysis. Data are expressed as mean ± SEM (*n* = 3 for protein analysis, *n* = 4–5 for gene expression analysis). * *p* < 0.05 versus CON; # *p* < 0.05 versus HF; † *p* < 0.05 versus HF + BP.

As increased levels of serum FFA from triacylglycerol induce hepatic toxicity by upregulating ER stress [31], we further examined ER stress-related protein and gene expressions. Phosphorylated JNK and eIF2α levels were significantly increased by the HF diet. The protein levels of activated JNK and its upstream transmembrane ER-residence stress sensor IRE1α were higher in the HF + BP group than in the HF group by 1.36-fold and 1.95-fold, respectively, although only JNK levels showed significant differences. Downstream members of other transmembrane ER-residence stress sensors, eIF2α and ATF4, showed lower expression levels in the HF + BP group than in the HF group. Notably, AM supplementation resulted in lower levels of t-IRE1α/HSC70, p-JNK/t-JNK, p-eIF2α/t-eIF2α, and ATF4/HSC70 by 2.43-fold, 1.85-fold, 1.61-fold, and 1.41-fold, respectively, compared to those of the HF + BP group (Figure 3a). In gene expression analysis, the levels of BiP and CHOP, which are mediators of ER stress induction, were higher in the HF + BP group than in the HF group by 1.63-fold and 1.65-fold, respectively. These increased expressions of BPA-aggravated ER stress induction were reduced by AM treatment, resulting in 0.58-fold and 0.67-fold reduction, respectively (Figure 3b). In addition, the expression of autophagy-related proteins was examined. Although HF feeding did not alter autophagy-related protein expression, 1.30-fold higher levels of p62, as well as 0.69-fold and 0.82-fold levels of LC3-II/LC3-I ratio and beclin-1, respectively, were detected in the HF + BP group compared to those in the HF group. In response to AM supplementation, p62 expression levels were decreased compared to those in the HF + BP group, but further effects on other proteins were not observed (Figure 3c).

### 3.4. Identification of Compounds in AM Extract by UHPLC-MS/MS and Molecular Network Analysis

**Figure 4:**
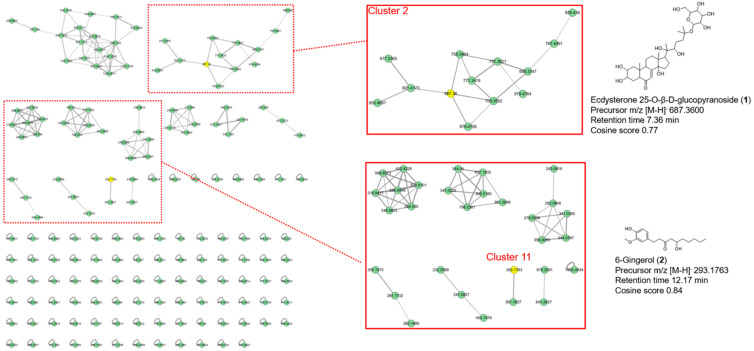
Feature-based molecular networking of the *Allium macrostemon* extract. A total of 162 nodes and 12 clusters (nodes ≥ 2) were organized into a network, and ecdysterone 25-O-β-D-glucopyranoside (1, Cluster 2) and 6-gingerol (2, Cluster 11) were identified.

As illustrated in Figure 4, 162 precursor ions were organized into a molecular network, which included 12 clusters (nodes ≥ 2) and 162 single nodes. Two compounds from the AM extract were identified as ecdysterone 25-O-β-D-glucopyranoside (1) in Cluster 2 and 6-gingerol (2) in Cluster 11 by the FBMN analysis; additional details can be found on the GNPS website (https://gnps.ucsd.edu/ProteoSAFe/status.jsp?task=d827b40931454be6bb67f9521e2a64c8 (accessed on 1 May 2023)). Compound **1** is identified as a new metabolite of ecdysone in the nematode Parascaris equorum [32], and 2 is known as a major bioactive component in ginger [33]. Compounds **1** and **2** have never been identified in *Allium macrostemon*.

## 4. Discussion

The primary aim of the present study was to evaluate the preventive effect of AM supplementation against the endocrine-disrupting chemical BPA in combination with HF-diet-altered adverse health effects. Although no difference in epididymal fat mass between the HF + BP group and HF group was observed, BPA suppressed HF diet-induced body weight gain. These results indicated that BPA may exert toxicity by inhibiting body weight gain. This result agrees with the results of a previous study, in which 8-week-old HF/high-cholesterol/high-cholic-acid diet (HFCCD)-fed C57BL/6 male mice were treated with 50 mg/kg/day BPA for 8 weeks [32]. BPA coupled with HFCCD increased body weight loss as a result of severe liver damage, as shown by the significantly increased level of liver injury serum marker, GPT, whose level was increased 2.23-fold compared to that in the HFCCD group. Here, we also determined serum liver injury marker levels, revealing modest increases in GOT and GPT levels in the HF + BP group compared to those in the HF group. In addition, no additional impact of BPA administration on epididymal fat mass in HF diet-fed mice indicates that lower body weight gain is not derived from lower adipose tissue weight. Previous studies have demonstrated that BPA-induced adverse health outcomes include bone loss [33]. It would be interesting to clarify the mechanism by which body weight loss occurs in future studies.

Similar to comparable weights, we observed no significant alterations of inflammation in the adipose tissue between the HF group and HF + BP group in our 9-week exposure period. This implies that there was no additional effect of BPA exposure in HF-induced adipose tissue inflammation. Similarly, previous studies also reported no adipose tissue mass difference between HF only and HF + BPA treatments in 4-week [34] or 12-week exposure schemes [35,36]. These previous studies proposed tissue-specific impacts of the HFD and BPA co-exposure condition. Although no mass alteration was seen in adipose tissue, brain inflammation [36] and skeletal muscle insulin signaling deterioration [35] were exaggerated in the co-exposure condition compared to only HF exposure. In line with previous studies, we observed an exaggerated harmful effect of additional bisphenol A exposure in terms of ER stress induction in liver tissue (Figure 3) as well as in testis tissue.

Activation of ER stress is a key pathological mechanism of steatosis [37]. Our data showed that HF + BP activated one ER branch, the IRE/JNK pathway, and inhibited the other branch, at the p-eIF2α/ATF4 level. Interestingly, ATF4 expression was significantly positively correlated with autophagy marker protein beclin-1 (r = 0.762, *p* = 0.004) and LC3-II/LC3-I (r = 0.611, *p* = 0.035) levels. These associations suggest that decreased autophagy in the HF + BP group may be linked to changes in the eIF2α/ATF4 pathway [13]. In a previous study using Neuro-2a cells, autophagy was decreased by BPA, as determined by the reduction in beclin-1 and LC3-II/LC3-I levels, suggesting BPA-induced neurotoxicity [38]. A macrophage study also showed a BPA-induced reduction in LC3-II/LC3-I levels and an increase in p62 levels, contributing to BPA-induced immune cell injury [39].

BPA’s exacerbation of dysfunction in the liver of mice fed with an HF diet was evident, as shown by the expression of certain ER stress markers, including p-JNK, CHOP, and BiP. Therefore, we propose that BPA’s exacerbation of dysfunction under the HF diet condition may be tissue-specific in our experimental model. Similar to our results, the aggravation effect of BPA on the HF diet in hepatic ER stress induction was reported in a rodent study of 3 months of BPA and HF diet co-exposure [32]. The differences in the experimental design, including a shorter treatment period (2 months in our study), sex, and species, may explain the inconsistent expression patterns of several other ER stress induction markers in our study compared to the previous report.

In this study, significant reduction in epididymal fat mass by AM supplementation against HF and BPA co-exposure was demonstrated. These results are consistent with those of a previous AM study [28]. In HF-diet-fed mice, daily administration of a steroidal saponin, macrostemonoside A, isolated from the bulbs of AM, at a concentration of 4 mg/kg for 30 days showed a decrease in serum TC levels and visceral fat accumulation [28]. In the present study, we used a dose of BPA of 50 μg/kg/day; this dose was set based on the dose considered safe for tolerable daily intake by the U.S. Environmental Protection Agency, and the dose was also tested in previous mouse studies [34,40]. The AM extract dose was also tested in a previous mouse study, with no side effects [41]. In human subjects with hyperglyceridemia, daily administration of a Chinese medicine extract of AM Xuezhitong at 2700 mg for 12 weeks induced a superior reduction in TC levels by 14.18% compared to 3.89% in the placebo group [28]. In addition, similar to the present results, a previous study demonstrated that administration of AM-derived saponins reduced visceral fat accumulation but was not accompanied by changes in body weight [28]. The lack of body weight reduction by AM may be explained by changes in body composition, including other fat depot alterations, as well as muscle weight, which warrants further study.

AM supplementation also improved adipose tissue dysfunction in the HF diet and BPA co-exposure condition. Similar to decreased epididymal fat pad weight, the reduction of inflammation, as determined by CLS and pro-inflammatory gene expression, was observed in the HF + BP + AM group compared to the HF group. In line with our data, a previous study investigating lycopene supplementation in a BPA-treated rats showed loss of adipose tissue weight and reduction of pro-inflammatory cytokine levels, explaining its antioxidant potential [16]. Similarly, lowered antioxidant enzyme expression levels were also observed in the HF + BP + AM group compared to the HF group. Previous studies have suggested that AM exhibits antioxidant and anti-inflammatory properties [24,26]. Nevertheless, since there was no difference in expression levels of inflammation and antioxidant markers between the HF group and the HF + BP group, the possibility of lowering adipose tissue dysfunction with regard to HF per se, rather than HF and BPA co-exposure, cannot be precluded.

We also demonstrated that AM reduced hepatic ER stress levels, although not all markers exhibited consistent alterations. The ameliorating effects of supplementation with stronger antioxidants, such as n-acetylcysteine, on HF plus BPA co-exposure has been reported previously [32]. To the best of our knowledge, amelioration of hepatic ER stress induction by AM extract was firstly reported in our study, with the possible mediator being the JNK pathway. The JNK pathway is known to mediate the induction of pancreatic ER stress by a major active liver metabolite of BPA, 4-methyl-2,4-bis(4-hydroxyphenyl)pent-1-ene [42]. Suppression of the JNK pathway by food components such as curcumin has been reported in a BPA-exposed liver cell model [5]. Still, the decrease in ER stress induction in the liver was not significant when compared to the control group. In summary, our study proved that AM supplementation can modify BPA-exaggerated ER stress induction in the liver, possibly through the JNK pathway, but the lowering effect is insufficient to completely mitigate the ER stress. Also, since the AM supplementation to the HF group was not tested, further study would be warranted to specify the AM’s ameliorating effect on the HF as well as the BPA exposure condition.

It is believed that the polyphenols in AM extract are responsible for its biological function. We previously reported the results of HPLC analysis showing that AM contains several phenolic compounds, including ellagic acid, protocatechuic acid, catechin, ferulic acid, chlorogenic, *p*-coumaric acid, and caffeic acid, which have anti-obesity, antioxidant, and anti-inflammatory activities [29]. Other major bioactive compounds, including steroidal saponins, flavonoids, phenylpropanoids, alkaloids, volatile oils, polysaccharides, and amino acids [23], were also reported from AM. In this study, we further analyzed AM using UHPLC-MS/MS and molecular networking analysis. The results suggest that AM supplementation can exert a preventative effect on the HF and BPA exposure condition. The analysis identified two compounds, named ecdysterone 25-O-β-D-glucopyranoside and 6-gingerol. Our results proposed identification of minor active compounds using nontargeted profiling analysis rather than NMR analysis, which mainly identifies major compounds that have been previously reported. Taken together, the various phytochemicals in AM extract, identified by two different analysis methods, might explain the beneficial effects, including anti-ER stress, antioxidant, and anti-inflammatory properties.

One of the limitations of our study was its relatively short duration of 9 weeks, which may have limited detection of significant negative effects on the liver and adipose tissue parameters induced by co-exposure of HF and BPA. Previous research reported that co-treatment of BPA for 3 months worsened hepatic steatosis in HF diet-fed mice [9], suggesting the need for longer testing periods in future studies to explore the role of supplementation in co-exposure scenarios. Another limitation of our study is that the number of animals used in this research was relatively small, although it was sufficient to demonstrate statistical differences between the groups. Nonetheless, the use of the minimum number of mice aligns with the ethical principles of the “Three Rs (Replacement, Reduction, and Refinement)” [43], which aims to minimize unnecessary pain and suffering to animals. Additionally, we did not include groups exposed to BPA only or BPA with AM supplementation. Despite this, we observed a significant ameliorating effect of AM in the HF + BP + AM group. The possibility of AM’s protective role against HF diet-induced effects cannot be precluded. Future research incorporating more groups or multiple dose provision could further elucidate the role of AM in countering the effects of an HF diet alone or in combination with BPA exposure.

## 5. Conclusions

Co-exposure to a high-fat diet and bisphenol A suppressed high-fat diet-induced body weight gain and hepatic endoplasmic reticulum stress induction. Although not all perturbations caused by co-exposure were recovered by *Allium macrostemon* extract supplementation, the *Allium macrostemon* extract–supplemented group showed reduced adipose tissue weight, adipose tissue inflammation, and hepatic endoplasmic reticulum stress levels compared to the non-supplemented high-fat + bisphenol A group. In the context of bisphenol A–exaggerated metabolic perturbations with a high-fat diet condition, the antioxidant potential of *Allium macrostemon* may play an important role in counteracting high-fat diet and bisphenol A–induced metabolic dysfunction in adipose tissue and liver. Further studies are needed to extend the evaluation of the effect of *Allium macrostemon* t against high-fat diet and bisphenol A co-exposure scenarios, which would highlight the necessity to develop a potential functional ingredient with *Allium macrostemon* to counteract the dietary issues in modern life.

## Figures and Tables

**Table 1:** Effects of *Allium macrostemon* (AM) extract on body weight gain, energy intake, organ weights, and serum analysis.

Parameters	CON	HF	HF + BP	HF + BP + AM
Body weight gain (g)	6.3 ± 0.5	16.6 ± 0.9 *^,#^	12.8 ± 0.7 *^,#^	12.9 ± 0.7 *^,#^
Energy intake (kcal/d/mouse)	8.94 ± 0.00	11.76 ± 0.72 *	11.47 ± 0.46 *	11.06 ± 0.21
				
Epididymal fat (g)	0.58 ± 0.06	2.09 ± 0.11 *	2.00 ± 0.06 *	1.57 ± 0.11 *^,#,†^
Perirenal and retroperitoneal fat (g)	0.29 ± 0.04	1.12 ± 0.07 *	0.87 ± 0.06 *	0.70 ± 0.11 *^,#^
Liver (g)	0.74 ± 0.03	0.80 ± 0.02	0.77 ± 0.02	0.74 ± 0.01
Kidneys (g)	0.26 ± 0.01	0.31 ± 0.02	0.27 ± 0.01	0.28 ± 0.01
Spleen (g)	0.06 ± 0.00	0.07 ± 0.00 *	0.06 ± 0.00 ^#^	0.06 ± 0.00 ^#^
				
Serum GOT (IU/L)	31.30 ± 7.08	21.78 ± 3.61	34.81 ± 7.39	46.74 ± 10.33
Serum GPT (IU/L)	24.50 ± 1.92	21.03 ± 0.72	31.71 ± 5.90	26.83 ± 3.08
Serum BUN (mg/mL)	18.22 ± 0.92	18.03 ± 0.44	17.03 ± 1.70	16.61 ± 0.95

Data are expressed as mean ± SEM (*n* = 6–7). * *p* < 0.05 versus CON; ^#^ *p* < 0.05 versus HF; ^†^ *p* < 0.05 versus HF + BP. CON, control diet group; HF, high-fat diet group; HF + BP, high-fat diet and bisphenol A–treated group; HF + BP + AM, high-fat diet and bisphenol A plus *Allium macrostemon* extract–treated group; GOT, glutamic oxaloacetic transaminase; GPT; glutamic pyruvic transaminase; BUN, blood urea nitrogen.

## Data Availability

The data presented in this study are available on request from the corresponding author.

## Supplementary Materials

The following supporting information can be downloaded at: https://www.mdpi.com/article/10.3390/foods12203777/s1. Table S1, Primer sequences used in quantitative real-time PCR.
