# Supplementary material for: Effects of Allium macrostemon Bunge Extract on Adipose Tissue Inflammation and Hepatic Endoplasmic Reticulum Stress in High-Fat Diet-Fed and Bisphenol A-Treated C57BL/6N Mice
Source: Foods. 2023 Oct 14;12(20):3777. doi: 10.3390/foods12203777 (PMC10606828; doi:10.3390/foods12203777)
Supplement: Supplementary file 1 [file foods-12-03777-s001.zip › foods-2595322-supplementary.pdf]

Supplementary Materials

Table S1. Primer sequences used in quantitative real-time PCR

**Gene**[1]		**Forward (5′→3′)**	**Reverse (5′→3′)**
Inflammation markers	*F4/80*	GGC CAT TGC CCA GAT TTT C	CGG TTG AGC AGA CAG TGA ATG A
	IL-1B	GCA ACT GTT CCT GAA CTC AAC T	ATC TTT TGG GGT CCG TCA ACT
	NOS2	CAG CTG GGC TGT ACA AAC CTT	CAT TGG AAG TGA AGC GTT TCG
	TNF-α	TGG CCT CCC TCT CAT CAG TT	CAG GCT TGT CAC TCG AAT TTT G
Antioxidant enzymes	*CAT*	TGG ACA AGT ACA ACG CTG AGA AG	AGC CGG CCT GCG TGT AG
	GPX	GGC TCA CCC GCT CTT TAC C	GGG TCG TCA CTG GGT GTT G
	SOD1	GGC CCG GCG GAT GA	GTC CTT TCC AGC AGT CAC ATT G
	SOD2	GCT GCA CCA CAG CAA GCA	CTC GGT GGC GTT GAG ATT G
	TRX2	CAG CCT CTG GCA CAT TTC CT	TGT TCG GCT TCT GGT TTC CT
ER stress markers	*GRP78/Bip*	ACC CTT ACT CGG GCC AAA TT	GCT TCA TGG TAG AGC GGA ACA
	CHOP	GCA TGA ACA GTG GGC ATC AC	CGA TGG TGC TGG GTA CAC TTC
Endogenous control	*18s rRNA*	AAC CCG TTG AAC CCC ATT	CCA TCC AAT CGG TAG TAG CG

[1] *F4/80*, adhesion G protein-coupled receptor E1; *IL-1β*, Interleukin-1β; *NOS2*, Nitric oxide synthase 2, inducible; *TNF-α*, Tumor necrosis factor-α; *CAT*, Catalase; *GPX*, Gluthatione peroxidase 1; *SOD1*, Superoxide dismutase 1, soluble; *SOD2*, Superoxide dismutase 2, soluble; *Trx2*, Thioredoxin 2; GRP78/Bip, 78-kDa glucose-regulated protein/binding immunoglobulin protein; *CHOP*, C/EBP homologous protein; *18s rRNA*, 18s ribosomal RNA.
